# Microtubule Integrity Is Associated with Mitochondrial Function and Quality of Murine Preimplantation Embryos

**DOI:** 10.3390/ijms26073268

**Published:** 2025-04-01

**Authors:** Yu-Ha Shim, Min-Jeong Cho, Min-Hee Kang, Yu-Jin Kim, Seung-A Oh, Ji-Soo Ryu, Byeong-Jun Mun, Jin-Young An, Jae-Ho Lee

**Affiliations:** 1 Department of Biomedical Science, College of Life Science, CHA University, Pocheon 11160, Republic of Korea; yuhaa525@gmail.com (Y.-H.S.); dhtmddk0102@gmail.com (S.-A.O.); jisuho5229@gmail.com (J.-S.R.); bjmun@chauniv.ac.kr (B.-J.M.); ajy6866@gmail.com (J.-Y.A.); 2 CHA Fertility Center, Seoul Station, Hangang-daero, Jung-gu, Seoul 04637, Republic of Korea; jminj725@chamc.co.kr (M.-J.C.); mhkang312@chamc.co.kr (M.-H.K.); yj_kim@chamc.co.kr (Y.-J.K.)

**Keywords:** embryo, cytoskeleton, microtubule, poor embryo quality, delivery ratio

## Abstract

Poor embryo quality is a major cause of poor clinical outcomes in assisted reproductive medicine, and there are no currently available interventions that can improve embryo quality. Mitochondria dysfunction is linked to low-quality female gametes and zygotes. Previously, microtubule integrity was also associated with mitochondrial function in oocytes. In the present study, we investigated the effects of the microtubule stabilizers (MTS) Taxol and Epothilone D (EpD) and the microtubule disturber (MTD) vinorelbine on mouse preimplantation embryo quality and pregnancy outcome compared with non-treatment controls. We prepared young BDF1 mice (7~9 weeks old) and cultured preimplantation embryos with MTS or MTD. Mitochondrial functional activity and embryo development ratios including pregnancy ratios were then assessed. MTS-treated embryos showed significantly increased mitochondrial membrane potentials and motility. Blastocyst formation was significantly higher in MTS-treated embryos than in MTD-treated embryos. Especially, MTS-treated embryos exhibited higher hatched blastocyte formation than untreated embryos. The number of offspring was significantly higher in surrogate mice transplanted with MTS-treated embryos. These findings demonstrated that the treatment of mouse preimplantation embryos with Taxol or EpD increased embryo development competence, which was associated with increased mitochondrial functional activity. Consistently, delivery ratios were significantly higher after transplantation with MTS-treated embryos than after transplantation with untreated embryos. These findings suggest that MTS could be used to supplement in vitro culture media to promote the recovery of poor-quality embryos.

## 1. Introduction

In vitro fertilization was introduced 40 years ago, and advances such as in vitro embryo maturation, intracytoplasmic sperm injection, vitrification, and preimplantation genetic testing have increased the success of in vitro fertilization. However, in vitro culture techniques and media formulations that improve poor-quality human oocytes and embryos are an unmet clinical need, and new approaches are currently being developed. No precise media formulations are available to optimize culture-to-blastocyst development ratios in poor-quality human embryos. Currently, the major causes of advanced-aged female infertility are embryo incompetence and low pregnancy outcomes in assisted reproductive technology (ART) [1,2,3,4]. However, no current interventions for age-related infertility can recover embryo competence for ART [1], and no specific embryo culture media formulations are available to improve embryo quality in ART for age-related infertility.

Embryo quality has been suggested to be highly associated with mitochondrial function. Several studies have reported that mitochondrial function can be improved by several agents, including anti-reactive oxygen species (ROS). However, mitochondrial dysfunction is still an unsolved issue for aged oocytes and embryos. Mitochondria are essential for the metabolism and catabolism of multiple substrates, the generation of metabolic signals, and the sensing of metabolic cues. Mitochondrial integrity affects cellular health, and mitochondrial defects are associated with numerous diseases [5]. However, therapeutic approaches that alleviate mitochondrial dysfunction are limited. Currently, the reversal of mitochondrial dysfunction is the focus of intense investigation, necessitating the identification of additional biochemical pathways that can be targeted to alleviate mitochondrial dysfunction [6]. However, mitochondria are dynamic structures that engage in fusion, fission, transport, and mitophagy. Although these behaviors are regulated by proteins distinct from mitochondrial bioenergetic reactions, recent studies suggest that metabolism and mitochondrial dynamics are tightly linked to significant inter-regulation [7]. Mitochondrial fusion and fission are unique processes that occur under different conditions to adapt mitochondrial morphology and dynamics to the cell’s bioenergetic requirements. Tau protein functional activity is linked to the net movement of axonal mitochondria to the neuronal cell body. Its dysfunction is more associated with dementia than beta-amyloid plaques. Microtubule integrity is regulated by the Tau protein, which affects mitochondria function and is required for the movement of mitochondria at the axons of neurons [8]. It performs this function by functioning as a linker protein between microtubule-associated proteins to stabilize microtubules. The Tau protein is an attractive target for new therapeutic approaches for dementia, and several small molecules that affect Tau function have entered clinical trials to examine their potential as treatments for neuronal degeneration disorders [9]. Because microtubule disintegration is considered a key cause of the mitochondrial dysfunction leading to dementia, reestablishing microtubule integrity could be a feasible therapy for dementia [10]. A previous study reported that a microtubule stabilizer promotes recovery from dementia [11].

In the oocyte, mitochondria are dynamic structures, and their degree of movement is associated with oocyte quality. Mitochondria move more slowly in aged oocytes than in young oocytes. We have previously reported that increased mitochondria movement in the oocyte may be associated with increased oocyte quality [12], suggesting that a small molecule microtubule stabilizer could be used to improve mitochondrial functional activity and thereby embryo quality and pregnancy success. The interplay between microtubule integrity and mitochondria functional activity increases embryo competence and pregnancy success. Oxidative damage such as that caused by ROS jeopardizes the mitochondrial genome during the aging process, which, together with microtubule instability, contributes to age-related mitochondrial dysfunction. We previously reported that cytoskeleton integrity dramatically decreases during aging, resulting in decreased mitochondrial functional activity and poor oocyte quality [8]. We also reported previously that actin cytoskeleton instability is possibly the primary cause of the loss of mitochondrial function in aged murine oocytes [8]. Mitochondrial motility and loss of function may be related to actin cytoskeleton instability during oocyte maturation in young and aged mice. Mitochondrial motility along the actin cytoskeleton may play a pivotal role in determining oocyte quality, depending on age. Also, studies using microtubule stabilizer-treated cells could reveal the relationship between microtubule integrity and mitochondria functional activity. However, the effects of microtubule stability on mitochondrial bioenergetics and function in embryo development are still unidentified, and the potential for microtubule stabilizers to stimulate these processes in embryo cultures has remained unexplored. Further, the regulatory mechanisms by which microtubule integrity supports mitochondrial function during embryonic development are unknown. The aim of the present study was to determine whether the use of a microtubule stabilizer would improve microtubule integrity to increase the bioenergetic and functional activities of mitochondria.

We also examined mitochondria functional activity in the preimplantation embryo and investigated whether two microtubule stabilizers (MTS), Taxol and Epothilone D (EpD), improve the competence of preimplantation embryo development and whether an MTS affects mitochondrial functional activity. As a negative control, we used embryos treated with vinorelbine (VNB), a microtubule disturber (MTD). Finally, we examined whether pregnancy ratios increased after the transplantation of microtubule stabilizer-treated embryos into the uterus.

## 2. Results

### 2.1. MTS Improved Embryo Development Ratios

**Figure 1:**
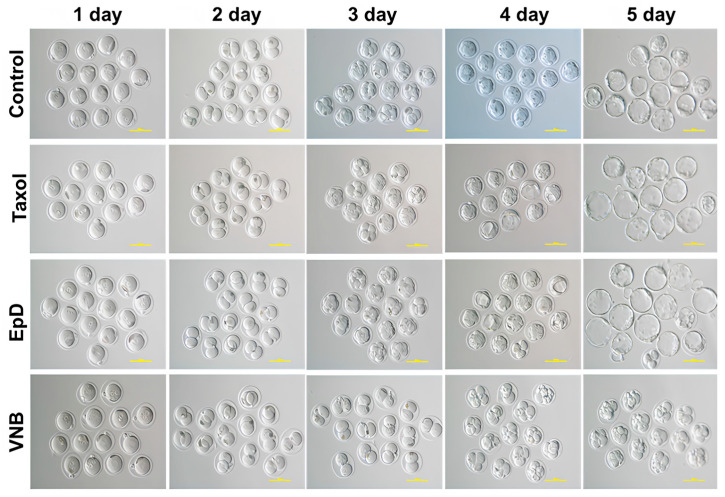
Preimplantation embryo development in embryos treated with an MTS or an MTD. Inverted microscope images of preimplantation embryo development of an in vitro fertilized embryo at 3.2~4.5 days after treatment with Taxol, EpD, or VNB. Scale bar = 200 um. Microtubule stabilizers (MTS) were Taxol and Epothilone D, and the microtubule disturber (MTD) was VNB.

**Figure 2:**
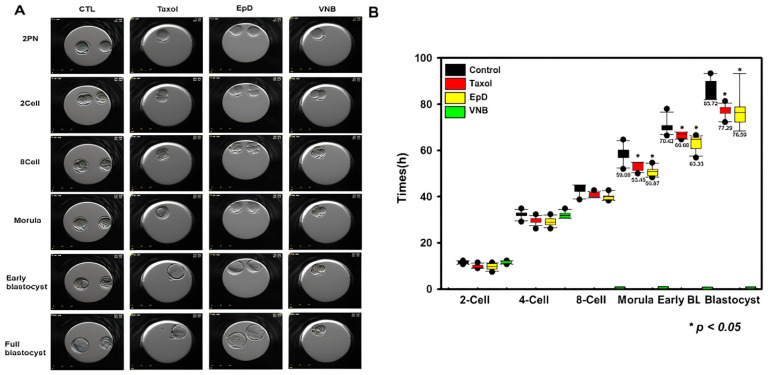
Developmental speeds of two-pronuclear (PN) fertilized embryos treated with an MTS (Taxol or EpD) or MTD (VNB) determined using a timelapse culture system. (**A**) Images of live embryos acquired using a timelapse culture system. (**B**) Development speeds of two-PN embryos to the blastocyst stage. Black bar, control embryos; red bar, Taxol-treated embryos; yellow bar, EpD-treated embryos; and green bar, VNB-treated embryos. Data are the mean ± standard error of the mean of three replicates (* *p* < 0.05 versus the control group).

The embryo development rate was higher in MTS-treated embryos than in control and MTD groups (Figure 1). The development ratios of MTS- and MTD-treated embryos were evaluated. The development speed was faster in MTS-treated embryos than in control and MTD-treated embryos (Figure 2). The times of each embryo development stage, including two-cell to four-cell, four-cell to eight-cell, eight-cell to morula, morula to blastocyst, and early blastocyst to late blastocyst, were decreased in MTS-treated embryos relative to the control and MTD-treated embryos (Figure 2). Blastocysts are more likely to implant and develop into successful pregnancies. Taken together, these findings demonstrate that MTS treatment increased the development rates of mouse embryos.

We measured the development speed of preimplantation embryos treated with MTS. Using a real-time cell monitoring system, the times at which embryos reached each developmental stage were measured (Figure 2B, Supplementary Video S1A (control embryos), Supplementary Video S1B (Taxol-treated embryos), Supplementary Video S1C (EpD-treated embryos), and Supplementary Video S1D (VNB-treated embryos)). Blastocysts in the groups treated with EpD (63 ± 13 h) and Taxol (68 ± 10.4 h) reached the fully expanded blastocyst stage faster than those in the control group (71 ± 11.4 h). The amount of time taken to reach the morula stage was dramatically decreased in the groups treated with EpD (51 ± 25.7 h) and Taxol (55 ± 23 h) relative to the control group (60 ± 22 h; Figure 2B). Furthermore, the transition from the morula stage to the late blastocyst stage occurred 9.09% and 18.18% faster in the EpD- and Taxol-treated groups than in the control group, respectively. Embryo development was arrested at the four-cell stage in the VNB-treated group.

Notably, preimplantation embryos treated with EpD and Taxol exhibited the fastest developmental speeds and highest blastocyst development rates. Treatment with an MTS improved embryo development, potentially by better recapitulating the microtubule integrity of preimplantation embryos.

### 2.2. Mitochondria Content and Membrane Potentials Were Higher in MTS-Treated Embryos

**Figure 3:**
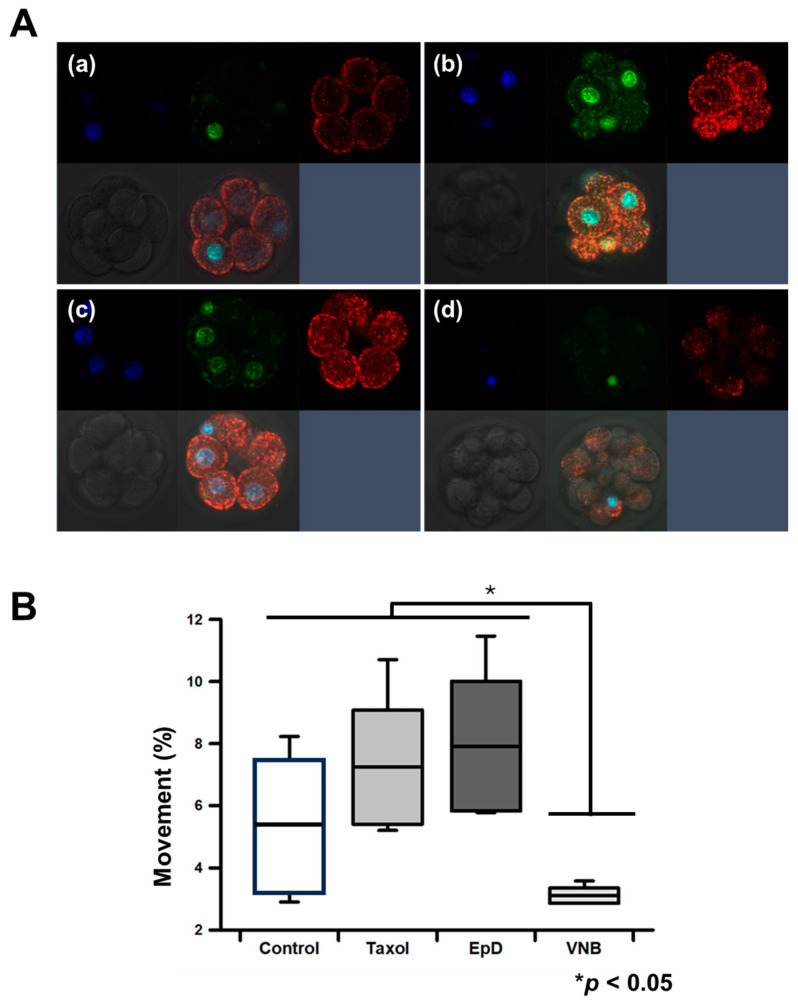
Mitochondria mass and membrane potential in eight-cell preimplantation embryos after treatment with an MTS or MTD. (**A**) Live confocal microscopy images of the preimplantation embryo labeled with MitoTracker Green (MTG) and MitoTracker Orange to measure mitochondria mass (green) and mitochondria membrane potential (ΔΨm) (red), respectively. Blue, nucleus staining with DAPI. (**a**) Control embryos, (**b**) Taxol-treated embryos, (**c**) EpD-treated embryos, and (**d**) VNB-treated embryos. (**B**) Quantification of mitochondrial moving ratios in eight-cell preimplantation embryos. Data are the mean ± standard error of the mean of three replicates (* *p* < 0.05 versus the VNB-treated group).

Mitochondria content and membrane potential were measured in MTS- and MTD-treated embryos using mitochondria-specific staining dyes, detecting mitochondria mass with MitoSpy Green FM and mitochondria membrane potential with MitoSpy Orange CMTMRos. Mitochondria content, indicated by a green signal, was significantly higher in MTS-treated embryos than in the control and MTD-treated embryos (Figure 3A). MTS also increased mitochondrial membrane potential relative to that in control and MTD-treated embryos (Figure 3B). Mitochondrial mass and membrane potential ratios were lowest in MTD-treated embryos.

### 2.3. Mitochondrial Dynamics in MTS- and MTD-Treated Embryos

Live confocal imaging was used to analyze the movement of mitochondria in preimplantation embryos (Figure 3). Microtubule staining was higher in MTS-treated embryos than in control or MTS-treated embryos (Figure 3A). Mitochondrial transport was faster in MTS-treated embryos (Figure 3(Ab,c) than in control embryos (Figure 3(Aa))). The maximum mitochondrial movement speed was 3 and 1 µm/s in MTS-treated and control embryos, respectively (Figure 3B, Supplementary Video S2A (control embryos), Supplementary Video S2B (Taxol-treated embryos), Supplementary Video S2C (EpD-treated embryos), and Supplementary Video S2D (VNB-treated embryos)). However, mitochondria movement was significantly lower in MTD-treated embryos than in MTS-treated embryos (Supplementary Video S2; A: control embryos, D: VNB-treated embryos). The average percentage of moving mitochondria, as detected by fluorescence staining, was measured under each experimental condition and was expressed as the percentage of mitochondria movement in each embryo.

### 2.4. Expression of Embryonic Genes in MTS- and MTD-Treated Embryos

**Figure 4:**
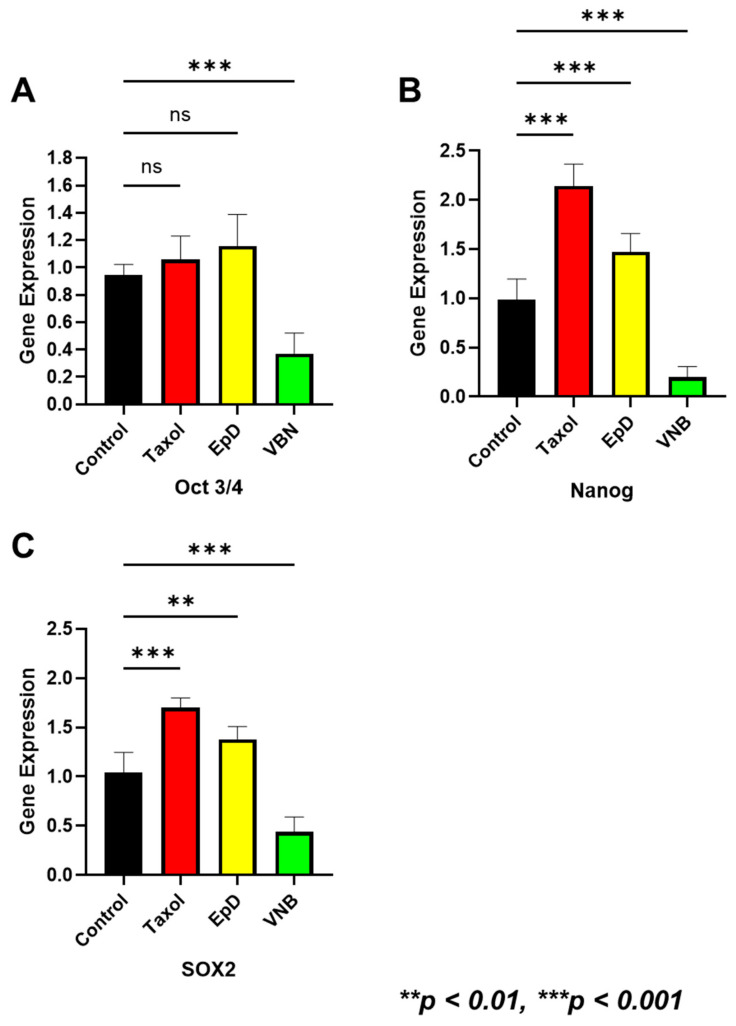
Expression of blastocyst formation genes in embryos treated with an MTS or an MTD. (**A**) Oct3/4, (**B**) Nanog, and (**C**) Sox2 expression. Data are the mean ± standard error of the mean of three replicates (ns indicates no statistically significant difference. ** *p* < 0.01 and *** *p* < 0.001 versus the control group).

Expression levels of Oct3/4, Nanog, and Sox2 were measured in MTS- and MTD-treated mouse blastocysts by RT-PCR (Figure 4). Oct3/4 mRNA levels were non-significantly increased in the MTS-treated group relative to those in the control and MTD-treated groups (Figure 4A). The mRNA levels of Nanog and Sox2 were significantly higher in MTS-treated blastocysts than in the control and MTD-treated blastocysts (Figure 4B,C).

### 2.5. Implantation and Delivery Ratios of MTS- and MTD-Treated Embryos

**Figure 5:**
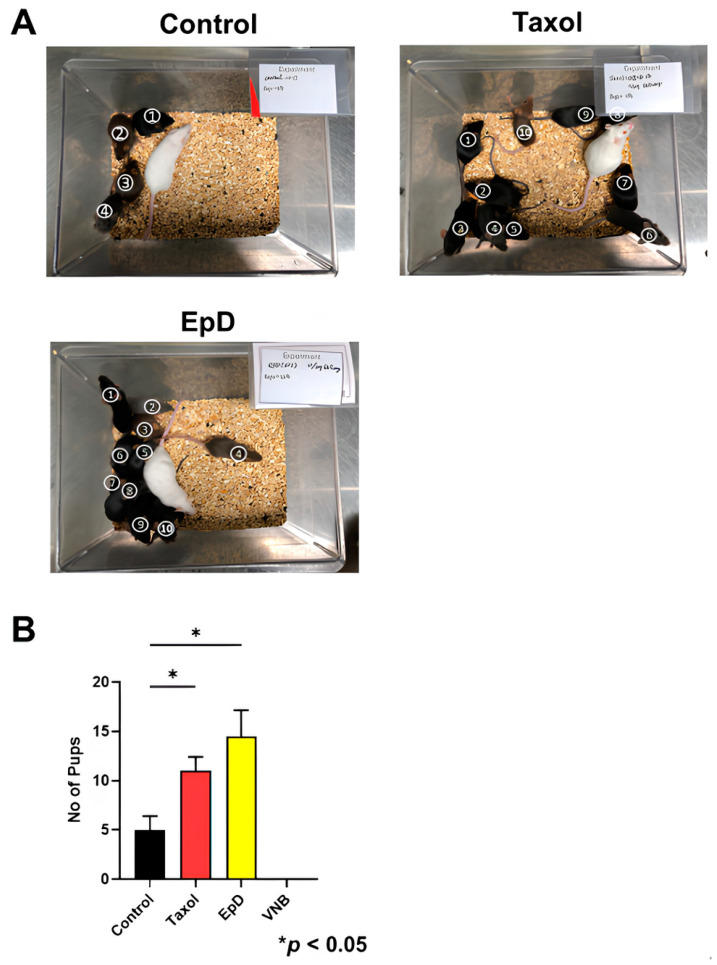
Offspring birth ratios of embryos with preimplantation MTS or MTD implanted into white recipient mice. (**A**) Offspring of MTS-treated embryos from BDF1 black mice conceived by implantation into ICR surrogate female mice. (**B**) Graph of delivery ratios from embryos treated with vehicle control, MTS, or MTD prior to embryo transfer (ET). Data are the mean ± standard error of the mean of three replicates (* *p* < 0.05 versus the control group). The number of pups per group is indicated in the figure.

The pregnancy and delivery ratios of EpD- and Taxol-treated embryos were significantly higher than those of the control group (Figure 5). Embryos treated with MTD (VNB) were not sufficiently competent for successful pregnancy or delivery of offspring. In MTS-treated embryos, those treated with EpD had significantly higher delivery ratios than embryos treated with Taxol.

## 3. Discussion

In the present study, we investigated the effects of the MTS (Taxol and EpD) on mouse embryonic development. MTS treatment during in vitro embryonic culture enhanced mitochondrial functional activity. Further, MTS significantly increased embryonic development and blastocyst ratios. Further, MTS treatment resulted in higher offspring delivery ratios than untreated control embryos. However, MTD-treated embryos were not sufficiently competent for implantation or successful pregnancy.

The MTS used in the present study is used in clinical practice as an anti-cancer therapy and neurodegenerative disease therapy. The anti-cancer mechanisms of the MTS Taxol are incompletely understood, but prior reports suggest that MTS target mitochondria-dependent apoptotic signaling [6]. However, the effects of these agents are dose-dependent. Therefore, Tau-targeted therapy has been introduced for the treatment of neurodegeneration disease [9]. Tau is a microtubule-associated protein that stabilizes microtubules, and its dysfunction causes neurodegeneration by reducing microtubule integrity. Several microtubule stabilizers are available, and some studies have shown that they are effective against Alzheimer’s Disease. Further, optimal doses of EpD, a microtubule stabilizer, improve cognition and decrease Tau pathology and Tau-related changes in mitochondria dynamics and quality [13].

In ARTs, the identification of rapidly developing embryos may be important because these embryos often have a higher chance of reaching the blastocyst stage and leading to a successful pregnancy. In this study, the development speeds of MTS-treated embryos from the 8-cell stage to the morula stage and from the morula stage to the blastocyst stage were faster than those of control embryos. The embryo development speed predicts various outcomes including complete blastocyst development, the implantation ratio, and the pregnancy ratio after ET. In particular, the morula stage is an important phase for initial differentiation from totipotent cells in blastomeres to the trophectoderm and the inner cell mass. The morula stage is shorter than the cleavage stage. In addition, the level of biological and molecular activity is high in the morula stage. Good-quality embryos develop faster than poor-quality embryos in ARTs. Morula compaction and regular blastocyst formation occurred significantly faster in mice that became pregnant than in those that did not. The development speed ratios are utilized to select good-quality embryos in order to achieve a successful pregnancy.

Previously, our group reported that mitochondrial motility along the actin cytoskeleton may play a pivotal role in determining oocyte quality depending on age [12]. The actin cytoskeleton is a system of filaments and fibers that are essential for survival and diverse cellular processes, and microtubules are key modulators that underpin these cellular processes [14]. In the actin cytoskeleton, microtubules regulate the mitochondrial trafficking of cargo proteins [15]. Also, they are not only involved in intercellular trafficking and movement but also in mitochondria quality control [15] in the preimplantation embryo. Microtubules are a major component of the actin cytoskeleton and are involved in essential processes such as chromosome segregation, structural support, and intracellular transport of vesicles and organelles such as mitochondria. Microtubule integrity is related to diverse biological activities such as cell signaling and organelle trafficking [15]. Also, microtubule rearrangement supports mitochondrial bioenergetics, which affect cell proliferation. Microtubule stability has been reported to promote the functional activity of mitochondria in oocytes [12]. These observations together with the results of the present study suggest that microtubule stability increases mitochondrial dynamics, embryo development ratios, and pregnancy rates. The integrity of the actin cytoskeleton is also essential for mitochondrial quality control in the preimplantation embryo.

We analyzed the gene expression of Oct4, Sox2, and Nanog in MTS-treated embryos. These are stemness factors and their high expression is indicative of good-quality embryos with a good development potential. In particular, Sox2 plays a pivotal role in inner cell mass formation and lineage segregation during the early stage of preimplantation embryo development [16]. MTS treatment enhanced Sox2 expression in embryos. Furthermore, MTS treatment enhanced the speed and quality of embryo development by regulating Oct4, Sox2, and Nanog gene expression.

We demonstrated that the stabilization of microtubules with Taxol and EpD increases embryo competence and promotes in vitro embryonic development. Therefore, cytoskeletal stability could affect mitochondrial motility and metabolic activity during embryonic development. The development ratios were higher in Taxol- and EpD-treated preimplantation embryos than in the control embryos. The development rate of fertilized embryos is an indicator of embryo quality [4]. MTS-treated embryos also have significantly higher blastocyte rates than untreated embryos. Faster embryonic development is associated with healthy metabolic activity and normal chromosome status. Prior reports of human preimplantation embryo timelapse cultures have demonstrated that early cleavage time and blastocyst formation are associated with increased ratios of euploid development and clinical pregnancy [17]. Our results demonstrate the positive effect of MTS-induced microtubule stability on blastocyst development and hatched-out ratios.

There have been several reports of an association between microtubule density and aging [8,18,19], and the abnormal regulation of microtubules has been linked to aging-related disorders, such as neurodegenerative disease [8,20]. Previously, we reported microtubule integrity in aged oocytes and embryos. Therefore, microtubule stability is also important for the recovery quality of aged oocytes and embryos. Microtubule integrity dramatically differs between young and old oocytes and mitochondrial functional activity is closely associated with oocyte and embryo quality [12,21]. Mitochondrial immobility reduces the amount of energy required for oocyte maturation. In general, dysfunctional mitochondria contribute to the aging process. Therefore, the recovery of functional mitochondria is important for ART with advanced-age oocytes and improved embryo quality. This is the first report to identify potential reagents for the recovery of functional mitochondria for preimplantation embryo development. Reagents for the recovery of functional mitochondria in embryos cultured in vitro have significant potential for ART used to overcome age-related infertility.

## 4. Materials and Methods

### 4.1. Mice and PN Embryo Collection

BDF1 hybrid female mice (7–9 weeks of age) and male mice (12 weeks of age) were purchased from Oriental Bio (Seongnam, Republic of Korea). Five females were used for embryo collection in a set of experiments, and each set of experiments was repeated three times. All procedures for animal breeding and care complied with the Institutional Animal Care and Use Committee (IACUC approval number: IACUC210117) regulations of CHA University (Pocheon, Republic of Korea). For in vivo hormone stimulation, follicle growth was promoted by injecting 7.5 IU PMSG (Pregnant Mare Serum Gonadotropin; RP1782725000; BioVendor, Brno, Czech Republic) into the abdominal cavity of female mice, and hyperovulation was induced by injecting 7.5 IU hCG (Human Chorionic Gonadotropin; 668900221; LG Chem, Seoul, Republic of Korea) after 48 h. For the second hormone injection, female mice were mated with male mice (1:1 = females–males). Two-PN embryos were collected 36 h after hCG injection and mating. In this experiment, 800 embryos were harvested from 40 mice.

### 4.2. Treatment and Culture of Two-PN Embryos with an MTS or MTD

Two-PN embryos were cultured with an MTS [2 pM Taxol (PHL89806; Sigma-Aldrich, St. Louis, MO, USA) or 2 pM EpD (ab143616; Abcam, Cambridge, UK)] or MTD [5 nM VNB (V2264-5MG, Sigma-Aldrich)]. The non-cytotoxic concentrations for the treatment of two-PN embryos were determined previously [22]. Embryos were cultured in individual wells in a timelapse system incubator (CNC Biotech, Suwon, Republic of Korea) with 5% CO_2_ at 37 °C in SAGE™ in vitro blastocyst media (ART-1029; Origio, Copenhagen, Denmark) supplemented with 10% Quinn’s Advantage™ serum protein substitute (ART-3011, Origio). Images were acquired every 5 min in each culture dish. Embryo development ratios and cleavage time points were quantified during timelapse culture. Embryo quality was classified based on cleavage speed and blastocyst ratio.

### 4.3. Confocal Microscopy Analysis of Mitochondrial Mass and Membrane Potential in Embryos

Timelapse live confocal microscopy of mitochondria in mouse preimplantation embryos was performed. To investigate mitochondrial membrane potential and mass, four-cell and eight-cell embryos were co-stained with 250 nM MitoSpy Orange CMTMRos (424803; BioLegend, San Diego, CA, USA), which localizes to mitochondria depending on membrane potential, and 250 nM MitoSpy Green FM (BioLegend), which labels mitochondria independent of membrane potential and can therefore be used to measure mitochondrial mass. Then, nuclei were stained with 1 µg/mL Hoechst 33342^®^ (H1399; Thermo Fisher Life Technologies, Carlsbad, CA, USA) for 5 min at room temperature. Thereafter, embryos were transferred to a droplet of medium on a confocal glass-bottom dish (SPL, Pocheon, Republic of Korea). Confocal microscopy images of live embryos were acquired using an LSM880 microscope with an Airyscan META device (Carl Zeiss AG, Oberkochen, Germany) in a covered live chamber system (5% CO_2_ and 37 °C). All timelapse live imaging involved capturing 100 images at an interval of 0.5 s. Each image was analyzed and exported as a moving file (25 frames/s) and a single picture (TIFF format) using ZEN2012 software (Carl Zeiss AG). Live imaging was performed with a confocal microscope to analyze mitochondria mass and membrane potential. Live images were evaluated using ImageJ (version 1.08) with the Difference Tracker plug-in to determine the dynamics of mitochondria in embryos. The moving files were used to calculate the average percentage of moving mitochondria and the maximum speed in the cytoplasm.

### 4.4. Gene Expression Analysis

Individual embryos were harvested for RNA isolation by aspiration into an injection pipette under negative pressure. Approximately 20 blastocyst embryos were pooled and sonicated for 30 s in a WiseClean sonicator (WUC-A10H; DAIHAN, Wonju, Republic of Korea) filled with ice. Subsequently, cDNA was synthesized with AccuPower^®^ CycleScript RT PreMix (K2050; Bioneer, Daejeon, Republic of Korea). Primers specific for mouse Oct3/4, Nanog, Sox2, and β-actin were used to amplify these target genes. PCR cycling conditions were as follows: 3 min at 95 °C followed by 50 cycles of denaturation for 10 s at 95 °C and annealing and extension for 20 s at 60 °C. The expression of each gene was normalized to that of β-actin. Real-time qPCR was performed using a real-time PCR machine (CFX Connect, 788BR08519; Bio-Rad, Hercules, CA, USA) and SYBR Green Supermix (172527, Bio-Rad). Primers were used to analyze the effects of MTS and MTD on gene expression in blastocyst embryos.

### 4.5. Assessment of Pregnancy and Birth Ratios

The reproductive capacities of MTS- and MTD-treated embryos were determined using BDF1 black mice. Mice were first prepared for pseudopregnancy receipt. Vasectomized male white ICR white mice and surrogate female white ICR mice (7–10 weeks of age) were used for ET studies. Female recipient ICR mice were mated with vasectomized male ICR mice overnight and sperm plugs were checked on the following day. Female mice with confirmed sperm plugs were used for ET. Blastocysts on day 5 of culture were implanted via direct intrauterine transfer using a 30-gauge needle.

In total, 5–7 blastocysts per culture condition were transferred into each uterine horn of a recipient ICR mouse. After ET, implantation and live birth ratios were compared between the control and experimental groups. Three mice were used for each type of treated blastocyst for each set of ET experiments, and the experiments were repeated three times for statistical analysis.

### 4.6. Statistical Analysis

All experiments were repeated in triplicate. Data are presented as average values (mean ± SD). Statistical analysis was performed using Sigma-Plot 12.5 software. A one-way ANOVA followed by a post hoc Holm–Sidak test was used to determine statistical significance. Significance levels were expressed as * *p* < 0.05, ** *p* < 0.01, and *** *p* < 0.001.

## 5. Conclusions

In conclusion, our data newly demonstrate the therapeutic potential of MTS such as Taxol and EpD, which could support the integrity of microtubules in preimplantation embryos. Microtubule stability is associated with embryo developmental competence between the morula and blastocyst stages. Conversely, a decrease in microtubule stability prevents embryo development at an early stage, such as the transition from the 2-cell stage to the 4-cell stage. An MTS increases mitochondrial movement and enhances mitochondrial function in embryos. Poor-quality human embryos exhibit characteristics similar to MTD-treated embryos. Microtubule stability is important for the development of preimplantation embryos. Therefore, in vitro embryo culture media could be supplemented with an MTS to improve embryo quality and development from the two-cell stage to the blastocyst stage.

## Data Availability

Data contained within the article.

## Supplementary Materials

The following supporting information can be downloaded at: https://www.mdpi.com/article/10.3390/ijms26073268/s1.
